# Phosphodiesterase 10A Inhibition Modulates the Corticostriatal Activity and L-DOPA-Induced Dyskinesia

**DOI:** 10.3390/ph15080947

**Published:** 2022-07-30

**Authors:** Rayanne Poletti Guimarães, Danilo Leandro Ribeiro, Keila Bariotto Dos Santos, Carlos Henrique Zanello Talarico, Lívea Dornela Godoy, Fernando E. Padovan-Neto

**Affiliations:** Department of Psychology, Faculty of Philosophy, Sciences and Letters of Ribeirão Preto, University of São Paulo, Ribeirão Preto 14040-901, SP, Brazil; rpoletti@alumni.usp.br (R.P.G.); daniloribeiro@usp.br (D.L.R.); keilabariotto@usp.br (K.B.D.S.); carlos.talarico@usp.br (C.H.Z.T.); liveagodoy@alumni.usp.br (L.D.G.)

**Keywords:** L-DOPA-induced dyskinesia, Parkinson’s disease, phosphodiesterase 10A, medium spiny neurons, corticostriatal pathway

## Abstract

The facilitation of corticostriatal transmission is modulated by the pharmacological inhibition of striatal phosphodiesterase 10A (PDE10A). Since L-DOPA-induced dyskinesia is associated with abnormal corticostriatal transmission, we hypothesized that inhibition of PDE10A would modulate L-DOPA-induced dyskinesia (LID) by regulating corticostriatal activity. 6-OHDA-lesioned rats were chronically treated with L-DOPA for one week. After that, for two additional weeks, animals were treated with the PDE10A inhibitor PDM-042 (1 and 3 mg/kg) one hour before L-DOPA. Behavioral analyses were performed to quantify abnormal involuntary movements (AIMs) and to assess the antiparkinsonian effects of L-DOPA. Single-unit extracellular electrophysiological recordings were performed in vivo to characterize the responsiveness of MSNs to cortical stimulation. The low dose of PDM-042 had an antidyskinetic effect (i.e., attenuated peak-dose dyskinesia) and did not interfere with cortically evoked spike activity. Conversely, the high dose of PDM-042 did not affect peak-dose dyskinesia, prolonged AIMs, and increased cortically evoked spike activity. These data suggest that the facilitation of corticostriatal transmission is likely to contribute to the expression of AIMs. Therefore, cyclic nucleotide manipulation is an essential target in controlling LID.

## 1. Introduction

The striatum is the largest structure in the basal ganglia and is directly involved with motor information processing. About 95% of the striatal neuronal population comprises projection neurons, named medium spiny neurons (MSNs) [1]. The main afferents that reach the striatum are glutamatergic projections coming from the cortex and thalamus (which exert excitatory activity on the MSNs) and dopaminergic projections from the substantia nigra compacta (SNc) [2]. The striatal output pathways are characterized by projections from the MSNs to the internal segment of the globus pallidus and substantia nigra reticulata (i.e., striatonigral or direct pathway) or the external segment of the globus pallidus (i.e., striatopallidal or indirect pathway). Neurodegeneration of dopaminergic neurons in the SNc results in a dopaminergic deficit in the striatum. The absence of dopamine in the striatum leads to an imbalance in the activities of the striatonigral and striatopallidal pathways, which, in turn, is responsible for the main motor symptoms of Parkinson’s disease (PD) [3,4].

L-DOPA is classically used as the “gold standard” PD treatment [5]. However, after 5 to 10 years of treatment with L-DOPA, about 75–80% of patients report the onset of L-DOPA-induced dyskinesia (LID) [6]. LID is characterized by casual, purposeless, and involuntary motor manifestations. When established, LID can become debilitating and even worse than the PD symptoms [7]. Regarding the pathophysiology, a recent study conducted in parkinsonian rats demonstrated that the firing rate of striatonigral MSNs increased, whereas the firing rate of striatopallidal MSNs decreased during the occurrence of LID [8]. Additionally, the optogenetic stimulation of striatonigral MSNs was sufficient to evoke LID in parkinsonian rats [8]. Thus, there is evidence that dopamine replacement with L-DOPA modulates striatal output pathways distinctly during LID. The glutamatergic cortical drive is also closely related to the pathophysiology of LID. Several studies using the magnetic resonance imaging (MRI) technique have demonstrated that cortical activity is hyperactivated or dysregulated in patients with PD and LID [9,10,11]. In particular, the primary motor cortex (M1) is likely to be an essential structure involved in the onset of LID [12]. Therefore, abnormalities in the glutamatergic corticostriatal drive to MSNs may contribute to the development of LID.

Drugs that interfere with the availability of cyclic nucleotides (cAMP and cGMP) were widely studied as potential therapeutic targets for treating LID and other movement disorders [13]. Cyclic nucleotides are essential second messengers critically involved in the modulation of corticostriatal glutamatergic transmission. By limiting the diffusion of cAMP/cGMP within subcellular domains of striatal MSNs, cyclic nucleotide phosphodiesterases (PDEs) enzymes modulate MSN excitability and short- and long-term glutamatergic corticostriatal transmission [14]. For instance, an early study found that cyclic nucleotide (cAMP and cGMP) levels decreased at peak incidence of LID in the cortex, striatum, and globus pallidus [15]. Studies using non-selective PDEs inhibitors such as zaprinast and UK-343664 decreased the incidence of LID in rodents [16,17]. More recently, the antidyskinetic potential of PDE10A inhibitors was investigated. The PDE10A inhibitor TP-10 was shown to attenuate LID in 6-OHDA-lesioned rats [18]. Additionally, studies performed in MPTP-treated monkeys demonstrated that a 5-week treatment with the PDE10A inhibitor MR1916 reduced LID without interfering with the antiparkinsonian effects of L-DOPA [19]. Further, MR1916 also reduced LID in the 6-OHDA-lesioned rat model of PD [20].

PDE10A is highly expressed in MSNs and is compartmentalized proximal to the plasma membrane of dendritic spines, putting it in position to regulate post-synaptic cyclic nucleotide signaling involved in the integration of glutamatergic and dopaminergic neurotransmission [21]. It was demonstrated that genetic deletion of PDE10A or inhibition of the enzyme with papaverine elicits behaviors associated with enhanced striatal output [22]. PDE10A inhibition was demonstrated to enhance membrane excitability and responsiveness of MSNs to cortical inputs [14,23,24,25]. Interestingly, this facilitative effect of PDE10A inhibition was eliminated by local infusion of the soluble guanylyl cyclase (sGC) inhibitor ODQ but not the adenylyl cyclase inhibitor SQ 22536. In line with this, the facilitatory effects of PDE10A inhibition were lost in neuronal nitric oxide (NO) synthase (nNOS)-knockout animals [24]. These results suggest that the facilitatory effects of PDE10A inhibition on corticostriatal transmission if strongly modulated by cGMP. Taken together, these results indicate that PDE inhibitors could be useful therapeutic agents in treating LID due to restoring abnormal glutamatergic costicostriatal transmission.

Striatonigral MSNs express dopamine D1 receptors that are positively linked to adenylyl cyclase. Activation of D1 receptors increases cAMP and augments the activity of striatonigral MSNs to enhance ongoing motor activity. Striatopallidal MSNs express D2 dopamine receptors that are negatively linked to adenyl cyclase. Dopamine activity on D2 receptors inhibits cAMP production and ongoing motor activity. Thus, timed boosts in dopamine release may promote motor activity by simultaneously enhancing striatonigral MSNs activity and suppressing striatopallidal MSNs activity. In PD, the reduced dopaminergic transmission results in attenuation of striatonigral MSNs activity and facilitation of striatopallidal MSNs activity, contributing to the generation of the motor symptoms of PD [26,27]. The hyperdopaminergic state in the dyskinetic striatum causes hyperactivity of striatonigral MSNs and hypoactivity of striatopallidal MSNs, contributing to the onset of abnormal involuntary movements [28]. Because PDE10A inhibition has an effect biased toward the activation of indirect pathway MSNs [14,29,30], it was suggested that the antidyskinetic effects of PDE10A inhibitors would emerge because of the enhancement of striatopallidal activity [18]. However, the specific mechanism related to the antidyskinetic effects of PDE10A inhibitors requires further investigation. Therefore, we hypothesized that inhibition of PDE10A would modulate LID by regulating corticostriatal activity. The current study used in vivo extracellular electrophysiological single-unit recordings to investigate the effects of PDE10A inhibition on LID on corticostriatal transmission in the 6-OHDA rat model of parkinsonism.

## 2. Results

### 2.1. Stimulating and Recording Electrode Placements 

In rat studies, all stimulating electrode tips were confirmed to lie in the frontal cortex between 3.2 and 4.2 mm anterior to the bregma, 1.4–2.5 mm lateral from the midline, and 1.0–3.0 mm ventral to the brain surface [31]. Placements for extracellular recording electrodes implanted into the striatum were verified to lie between 1.2 mm anterior and 0.26 mm posterior from the bregma, 1.8–3.9 mm lateral from midline, and 3.7–5.6 mm ventral to the brain surface [31]. 

### 2.2. Dopaminergic Lesion Assessment

**Figure 1:**
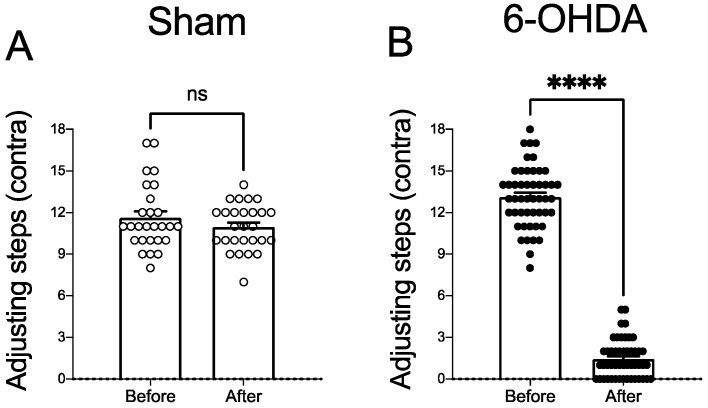
Evaluation of the stepping test before and after surgery for the unilateral vehicle or 6-OHDA infusion into the right MFB. (**A**) Administration of the vehicle into the right MFB bundle did not affect the stepping test (*p* > 0.05; paired *t*-test). (**B**) 6-OHDA microinjection into the right MFB significantly decreased the number of adjusting steps performed with the contralateral forelimb (**** *p* < 0.0001; paired *t*-test). Data expressed as mean ± SEM. Results are derived from a total of *n* = 23 Sham-operated and *n* = 51 6-OHDA-lesioned rats.

The stepping test was conducted to evaluate the akinesia of the contralateral forelimb to the lesion and select putative 6-OHDA-lesioned animals [32,33]. A paired-samples *t*-test was performed to compare the number of adjusting steps before and 4 weeks after vehicle or 6-OHDA microinjection in all animals used in this study. Vehicle microinjection into the medial forebrain bundle (MFB) did not affect the number of contralateral adjusting steps 4 weeks after the surgery (*t*_(26)_ = 1.90, *p* > 0.05; Figure 1A). There was a significant decrease in the number of steps contralateral to the lesion of the animals after surgery (*t*_(48)_ = 30.31, *p* < 0.0001; Figure 1B). All animals in this study presented a severe (>90%) reduction of TH immunoreactivity in the ipsilateral substantia nigra compacta.

### 2.3. L-DOPA Facilitates Corticostriatal Transmission in Dyskinetic Animals

#### 2.3.1. AIMs and Stepping Test

**Figure 2:**
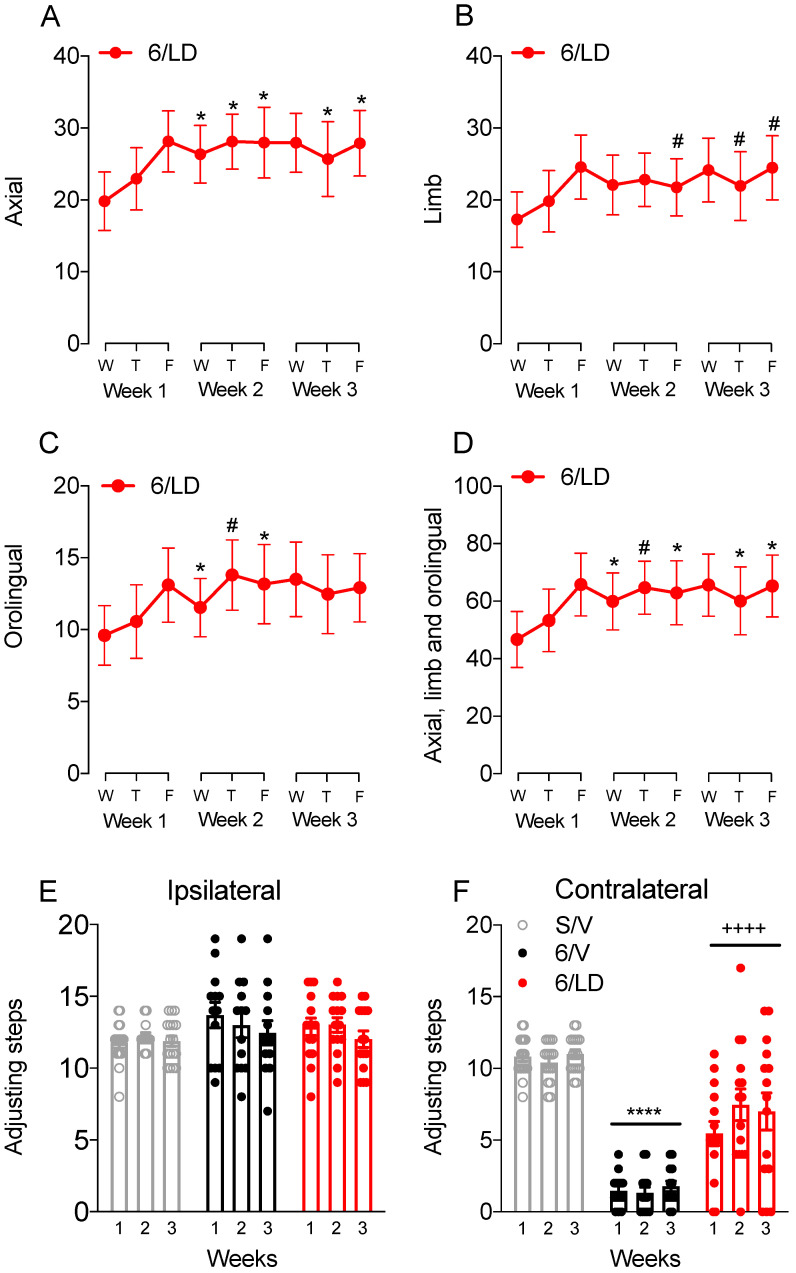
Effects of chronic L-DOPA administration on AIMs and stepping test. The temporal profile of (**A**) axial, (**B**) limb, (**C**) orolingual, and (**D**) sum of axial, limb, and orolingual AIMs is detailed for each week of chronic L-DOPA treatment. The analysis revealed that L-DOPA increased the incidence of AIMs throughout chronic administration (^#^ *p* < 0.10, * *p* < 0.05 vs. Wednesday on Week 1, one-way RM-ANOVA and Holm-Sidak post-hoc test). L-DOPA had no effect on the stepping test performed with the (**E**) ipsilateral forelimb and partially improved stepping test performance with the (**F**) contralateral forelimb (**** *p* < 0.001 vs. S/V group, ^++++^ *p* < 0.001 vs. 6/V group). Data expressed as mean ± SEM. Results are derived from n=17 animals in the S/V group, *n* = 13 in the 6/V group, and 15 in the 6/ LD group. Abbreviations: W = Wednesday, T = Thursday, F = Friday.

The behavioral analysis demonstrated that chronic administration of L-DOPA increased axial, limb, and orolingual AIMs in the 6-OHDA-lesioned rat model of PD. One-way RM-ANOVA revealed the significant effect of the L-DOPA treatment on the incidence of total axial (*F*_(8,112)_ = 3.02, *p* < 0.01; Figure 2A), orolingual (*F*_(8,112)_ = 3.03, *p* < 0.01; Figure 2C), and in the sum of axial, limb and orolingual AIMs (*F*_(8,112)_ = 3.10, *p* < 0.01; Figure 2D). There was a trend towards a significant effect on the incidence of limb AIMs (*F*_(8,112)_ = 1.90, *p* = 0.065; Figure 2B). Post-hoc comparisons revealed that chronic administration of L-DOPA increased the incidence of AIMs compared to the first scoring day (*p* < 0.05 vs. Wednesday on week 1). 

The stepping test performance was used as an indicator of the antiparkinsonian effect of L-DOPA. Two-way RM-ANOVA revealed no effect for drug treatment (*F*_(2,42)_ = 1.27; *p* > 0.05), time (*F*_(2,84)_ = 2.86; *p* > 0.05), and no interaction between drug treatment and time (*F*_(4,84)_ = 1.10; *p* > 0.05) on the number of adjusting steps performed with the ipsilateral side (Figure 2E). On the side contralateral to the lesion (Figure 2F), two-way RM-ANOVA revealed a significant main effect of drug treatment (*F*_(2,42)_ = 75.54; *p* < 0.0001). There was no effect of time (*F*_(2,84)_ = 1.18; *p* > 0.05), and no interaction between drug treatment and time (*F*_(4,84)_ = 1.48; *p* > 0.05). Post-hoc comparisons for the main effects of drug treatment revealed that L-DOPA partially restored akinesia in parkinsonian animals (*p* < 0.001 vs. S/V and 6/V groups).

#### 2.3.2. Electrophysiological Recordings in Cortically Responsive MSNs

The same cohort of animals/treatment groups described above was used for in vivo extracellular recording studies. All animals continued on the same daily treatment regimen following termination of behavioral studies on week 3 and were used in electrophysiological studies on vehicle/drug treatment on week 4. Electrophysiological recordings were in multiple putative striatal MSN neurons before (“off drug”) and 30–180 min after the last injection of vehicle/drug (“on drug”). Because the numbers of identified striatonigral MSNs were not sufficient for statistical comparisons, data from striatonigral (identified via antidromic stimulation of the SNr) and unidentified MSNs were pooled. 

**Figure 3:**
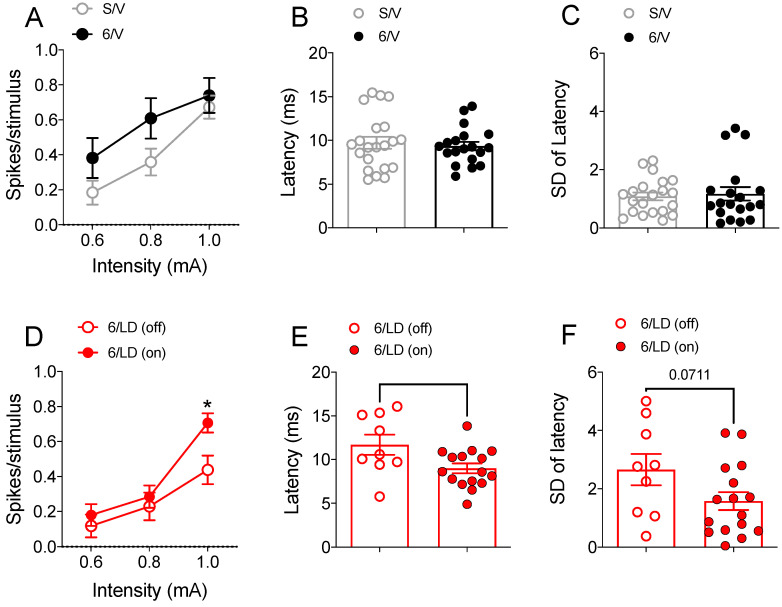
Corticostriatal signaling is altered in LID. (**A** to **C**) Current input/spike output relationship, latency to the first spike, and standard deviation of latency to the first spike observed in MSNs recorded in sham-operated and 6-OHDA-lesioned rats chronically treated with vehicle. (**A**) There was an interaction between the lesion and current intensity in the total number of spikes evoked per cortical stimulus between 6-OHDA-lesioned and sham-operated animals (*p* < 0.05, two-way RM-ANOVA) but no differences in post-hoc analysis (*p* > 0.05, Holm-Sidak). (**B**) Measures conducted at 1 mA cortical stimulation revealed no differences in the latency to the first spike (*p* > 0.05, *t*-test) or in the (**C**) standard deviation of the latency to the first spike (*p* > 0.05, *t*-test) between sham-operated and 6-OHDA-lesioned animals. (**D** to **F**) Current input/spike output relationship, latency to the first spike, and standard deviation of latency to the first spike observed in MSNs recorded in 6-OHDA-lesioned dyskinetic rats before (off) and after (on) L-DOPA administration. (**D**) Analysis of the total number of spikes evoked per cortical stimulus revealed that L-DOPA treatment facilitated cortically evoked responses (* *p* < 0.05 vs. 6/LD (off) at 1 mA, two-way RM-ANOVA and Holm-Sidak post-hoc test). (**E**) L-DOPA shortened the latency to the first spike (* *p* < 0.05 vs. 6/LD (off), *t*-test) and (**F**) reduced the standard deviation of latency to the first spike (*p* = 0.071 vs. 6/LD (off), *t*-test). Data are presented as the mean ± SEM. Results are derived from *n* = 10/21 animals/cells in the S/V group, *n* = 6/19 animals/cells in the 6/V group, and 12/9–16 animals/cells in the 6/ LD group.

We first determined the impact of 6-OHDA lesions on corticostriatal transmission (Figure 3A–C). Two-way RM-ANOVA revealed an interaction between lesion and current intensity (*F*_(2,76)_ = 4.89, *p* < 0.05, Figure 3A). However, the post-hoc analysis did not indicate differences in the total number of spikes evoked per cortical stimulus between 6-OHDA-lesioned and sham-operated animals across different current intensities applied to the motor cortex (*p* > 0.05). Because cortical stimulation with 0.6 mA (and sometimes 0.8 mA) failed to elicit consistent responses in MSNs, we further investigated the effects of cortical stimulation at 1 mA current intensity. Measures conducted at 1 mA cortical stimulation revealed no differences in the latency to the first spike (*t*_(38)_ = 0.44, *p* > 0.05; Figure 3B) or in the standard deviation of the latency to the first spike (*t*_(38)_ = 0.32, *p* > 0.05; Figure 3C) between sham-operated and 6-OHDA-lesioned animals. We then compared the impact of L-DOPA administration on corticostriatal transmission in dyskinetic animals (Figure 3D–F). Two-way RM-ANOVA revealed an interaction between drug treatment and current intensity (*F*_(2,46)_ = 3.36, *p* < 0.05, Figure 3D). Post-hoc analysis revealed that L-DOPA facilitated corticostriatal transmission at the 1mA current intensity applied to the motor cortex (*p* < 0.05 vs. “off” L-DOPA). Other measures conducted at 1 mA cortical stimulation revealed that L-DOPA reduced the latency to the first spike (*t*_(23)_ = 2.40, *p* < 0.05 vs. “off” L-DOPA; Figure 3E). Additionally, there was a trend toward a reduction in the standard deviation of the latency to the first spike (*t*_(23)_ = 1.89, *p* = 0.071 vs. “off” L-DOPA; Figure 3F).

**Figure 4:**
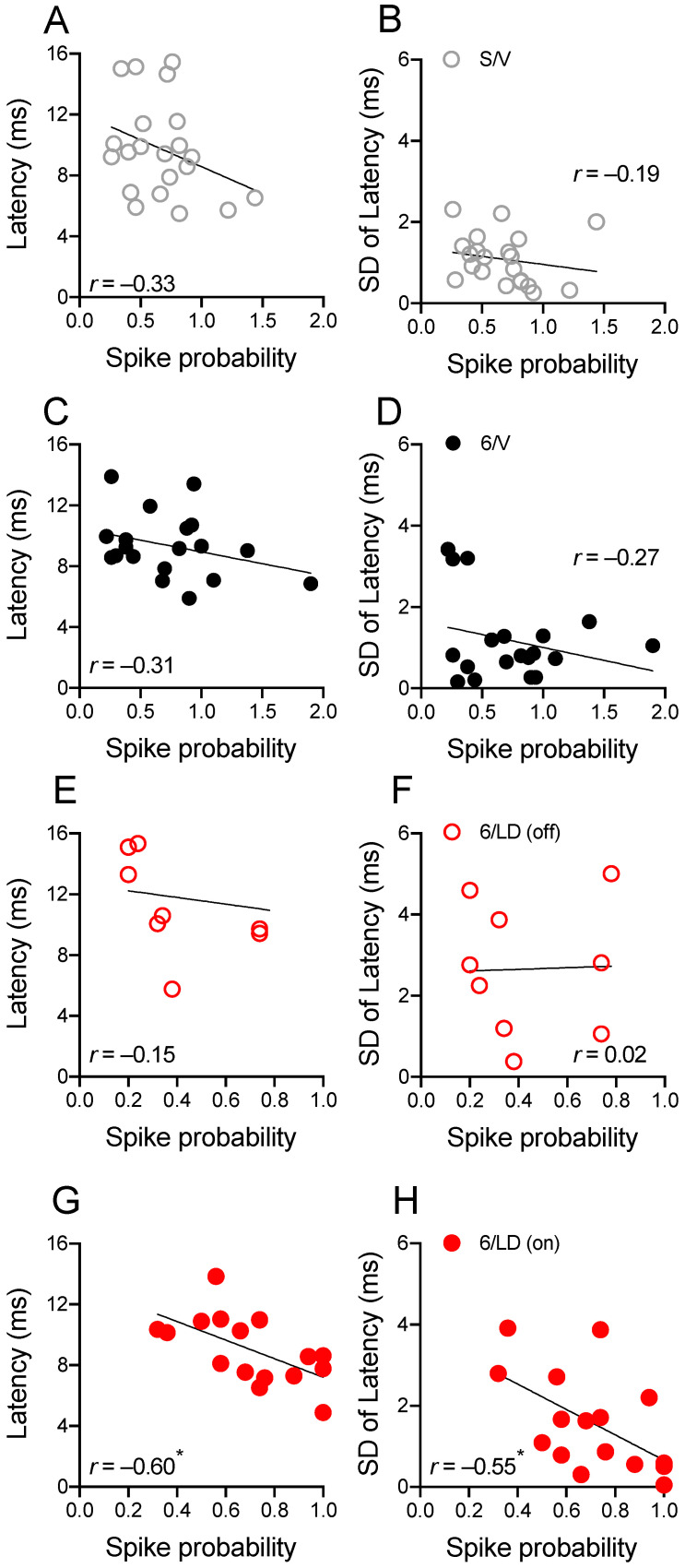
Spike probability and the latency to the first spike correlate in LID. (**A**–**F**) There was no correlation between the latency to the first spike and spike probability or the standard deviation of latency to the first spike and spike probability in the S/V, 6/V, and 6/LD groups (*p* > 0.05, Pearson’s *r*). (**G**) There was a negative correlation between the latency to the first spike and spike probability and (**H**) a negative correlation between the latency to the first spike and spike probability (* *p* < 0.05, Pearson’s *r*). Results are derived from *n* = 10/21 animals/cells in the S/V group, *n* = 6/19 animals/cells in the 6/V group, and 12/9-–16 animals/cells in the 6/ LD group.

The effects of L-DOPA treatment on spike probability and the latency to the first spike following cortical stimulation at 1 mA current intensity were further investigated with correlation analysis (Figure 4). There was no correlation between the latency to the first spike and spike probability (*p* > 0.05) or the standard deviation of latency to the first spike and spike probability (*p* > 0.05) in the S/V (Figure 4A,B) and 6/V (Figure 4C,D) groups. Interestingly, after L-DOPA administration (but not before; Figure 4E,F, *p* < 0.05), there was a significant negative correlation between the latency to the first spike and spike probability (*p* < 0.05; Figure 4G). Further, there was a negative correlation between the latency to the first spike and spike probability (*p* < 0.05; Figure 4H).

#### 2.3.3. Electrophysiological Recordings in Spontaneously Active MSNs

**Figure 5:**
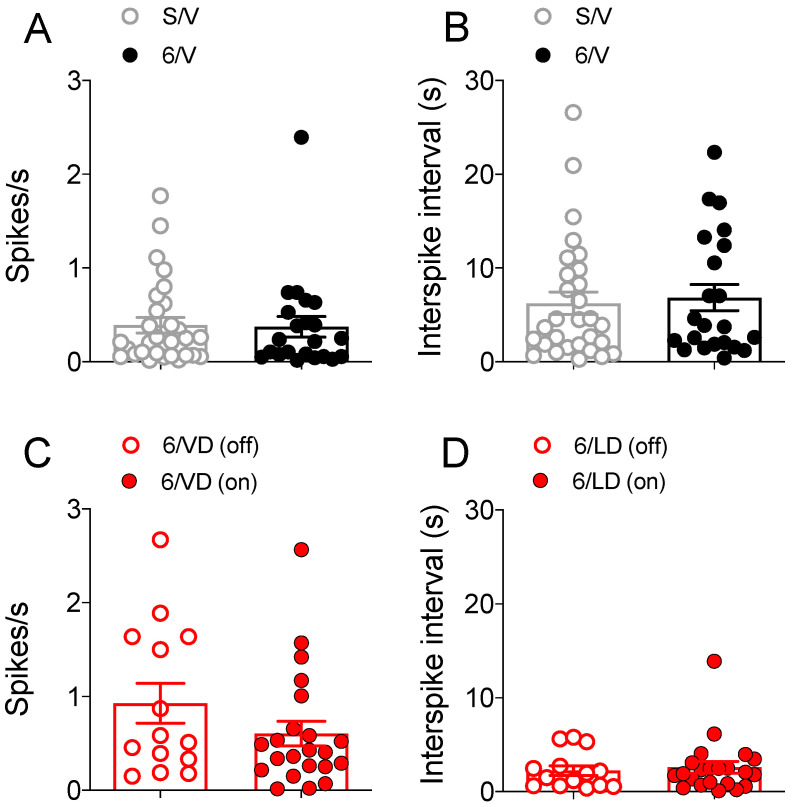
Group comparisons of the 6-OHDA lesion and L-DOPA treatment on spontaneous activity recorded from spontaneously active unidentified striatal MSNs. There was no difference in the (**A**) firing rate and (**B**) interspike interval in MSNs recorded in the striatum of sham-operated and 6-OHDA-lesioned rats (*p* > 0.05, *t*-test). There was no difference between (**C**) firing rates or (**D**) interspike interval in MSNs recorded in the striatum of dyskinetic rats before (off) and after (on) L-DOPA administration (*p* > 0.05, *t*-test). Data are presented as the mean ± SEM. Results are derived from *n* = 14/29 animals/cells in the S/V group, *n* = 10/22 animals/cells in the 6/V group, and 13/14–22 animals/cells in the 6/LD group.

The firing rate and the interspike interval were recorded in spontaneously active putative MSNs (Figure 5). There was no difference between firing rates (*t*_(49)_ = 0.13, *p* > 0.05; Figure 5A) or interspike interval (*t*_(49)_ = 0.32, *p* > 0.05; Figure 5B) of spontaneously firing striatal MSNs in sham-operated and 6-OHDA-lesioned groups. Although firing rate was higher and interspike interval was shorter in the 6/LD group (compared to S/V and 6/V groups), there was no difference between firing rates (*t*_(34)_ = 1.37, *p* > 0.05; Figure 5C) or interspike interval (*t*_(34)_ = 0.38, *p* > 0.05; Figure 5D) of spontaneously firing striatal MSNs recorded before (off) L-DOPA and after (on) L-DOPA administration.

### 2.4. Effects of Chronic Administration of the PDE10A Inhibitor PDM-042 on AIMs

#### 2.4.1. The Effects of PDM-042 on AIMs Are Dose-Dependent

**Figure 6:**
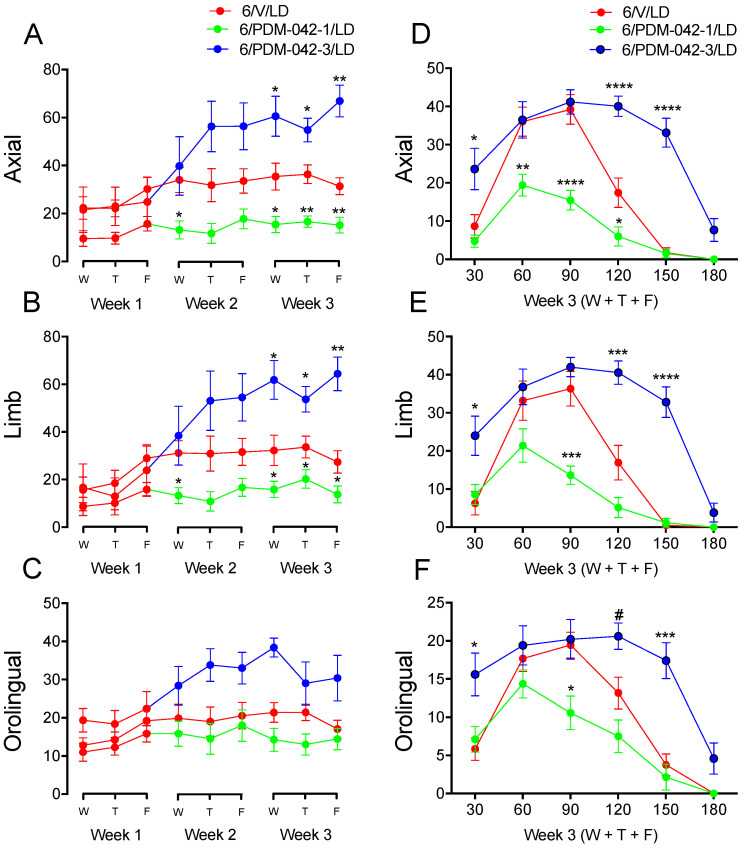
The effects of chronic administration of the PDE10A inhibitor PDM-042 on L-DOPA-induced AIMs are dose-dependent. The analysis of total axial (**A**), limb (**B**), and orolingual (**C**) AIMs revealed that the lower dose of PDM-042 (1 mg/kg) attenuated, whereas the higher dose (3 mg/kg) increased the incidence of AIMs (* *p* < 0.05, ** *p* < 0.01 vs. V/LD group, two-way RM-ANOVA and Holm-Sidak post-hoc test). The temporal analysis for the incidence of axial (**D**), limb (**E**), and orolingual (**F**) AIMs was investigated during the third week of chronic treatment. Scores applied at 180 min were excluded from the statistical analysis because most animals did not display AIMs at this time. However, this data point is still indicated in the chart. The analysis revealed that the lower dose of PDM-042 (1 mg/kg) attenuated AIMs during the peak effect of L-DOPA (60 to 120 min). The higher dose of PDM-042 (3 mg/kg) did not interfere with AIMs scored from 60 to 120 min but prolonged the peak-effect of L-DOPA further to 120 and 150 min (^#^ *p* = 0.09, * *p* < 0.05, ** *p* < 0.01, *** *p* < 0.001, **** *p* < 0.001 vs. V/LD group, two-way RM-ANOVA and Holm-Sidak post-hoc test). Data expressed as mean ± SEM. Results are derived from *n* = 9 animals in the 6/V/LD group, *n* = 7 animals in the 6/PDM-042-1/LD group, and 5 animals in the 6/PDM-042-3/LD group.

The second cohort of animals was used to investigate the effects of the PDE10A inhibitor PDM-042 on the incidence of AIMs. The results demonstrated that the effects of PDM-042 on AIMs were dose-dependent. Two-way RM-ANOVA revealed significant interactions between time and drug treatment for the analysis of total axial (*F*_(16,144)_ = 3.83, *p* < 0.0001; Figure 6A) and limb AIMs (*F*_(16,144)_ = 3.69, *p* < 0.0001; Figure 6B). Post-hoc comparisons revealed that chronic administration of the PDM-042 at a lower dose (1 mg/kg) attenuated axial (Figure 6A) and limb (Figure 6B) AIMs at the second (Wed) and third (Wed, Thu, and Fri) week of chronic treatment (*p* < 0.05 vs. V/LD group). Chronic administration of the PDM-042 at a higher dose (3 mg/kg) increased axial (Figure 6A) and limb (Figure 6B) AIMs during the third week (Wed, Thu, and Fri) of chronic treatment (*p* < 0.05 vs. V/LD group). Although there was no significant interaction between time and drug treatment for the analysis of total orolingual AIMs (*p* > 0.05; Figure 6C), there was a significant main effect of treatment (*F*_(16,144)_ = 1.36, *p* < 0.001). Post-hoc comparisons for main effects revealed that PDM-042 1 mg/kg did not interfere (*p* > 0.05 vs. V/LD group) with orolingual AIMs but PDM-042 3 mg/kg increased orolingual AIMs (*p* < 0.05 vs. V/LD group).

The temporal incidence for each AIM subtype was further investigated over the third week of chronic treatment (Figure 2D–F). Two-way RM-ANOVA revealed significant interactions between time and drug treatment for the temporal analysis of axial (*F*_(8,72)_ = 7.46, *p* < 0.0001; Figure 6D), limb (*F*_(8,72)_ = 6.10, *p* < 0.0001; Figure 6E), and orolingual AIMs (*F*_(8,72)_ = 5.63, *p* < 0.0001; Figure 6F). Post-hoc comparisons revealed that the lower dose of PDM-042 (1 mg/kg) attenuated axial (Figure 6D), limb (Figure 6E), and orolingual (Figure 6F) AIMs during the peak effect of L-DOPA (60 to 120 min, *p* < 0.05 vs. V/LD group). The higher dose of PDM-042 (3 mg/kg) increased axial (Figure 6D), limb (Figure 6E), and orolingual (Figure 6F) AIMs at 30 min post-L-DOPA administration (*p* < 0.05 vs. V/LD group). Further, PDM-042 3 mg/kg prolonged the peak effect of L-DOPA at 120 and 150 min (*p* < 0.05 vs. V/LD group). Interestingly, PDM-042 at 3 mg/kg did not increase AIMs at the 60 and 90 min post-L-DOPA injection mark (*p* > 0.05 vs. V/LD group). These results suggest the higher dose of the PDE10A inhibitor prolongs rather than exacerbates L-DOPA-induced AIMs.

**Figure 7:**
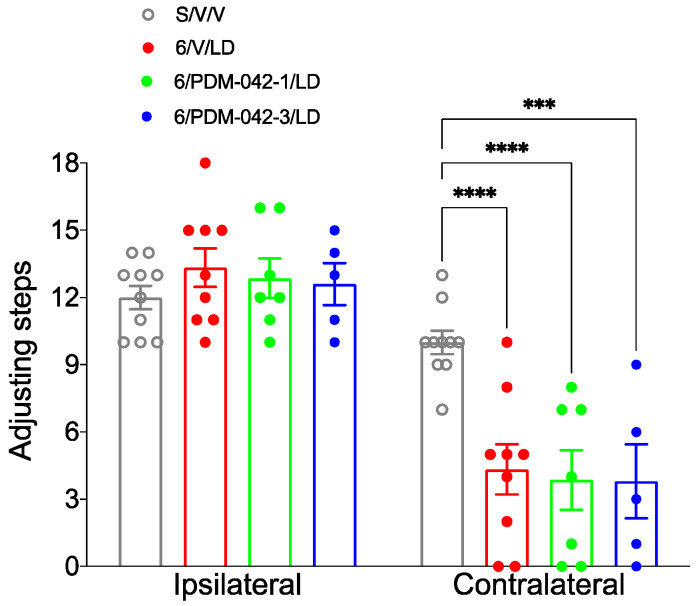
Effects of chronic administration of the PDE10A inhibitor PDM-042 on the stepping test. Low (1 mg/kg) and high (3 mg/kg) doses of PDM-042 did not interfere with the number of adjusting steps performed with the ipsilateral forelimb. Additionally, PDM-042 did not interfere with the antiparkinsonian activity of L-DOPA (*** *p* < 0.01, **** *p* < 0.01 vs. S/V/V group, two-way RM-ANOVA and Holm-Sidak post-hoc test). Data expressed as mean ± SEM. Results are derived from *n* = 9 animals in the 6/V/LD group, *n* = 7 animals in the 6/PDM-042-1/LD group, and 5 animals in the 6/PDM-042-3/LD group.

The stepping test was performed on the third week of chronic treatment to evaluate whether PDM-042 interferes with the antiparkinsonian activity of L-DOPA (Figure 7). Two-way RM-ANOVA revealed an interaction between the drug treatment and forelimb side (contralateral and ipsilateral) (*F*_(3,27)_ = 6.48, *p* < 0.01). The post-hoc analysis revealed no interference of drug treatment on the number of adjusting steps performed with the ipsilateral forelimb (*p* > 0.05, Figure 7, left). The post-hoc analysis also revealed a partial antiparkinsonian activity of L-DOPA on the number of adjusting steps performed with the contralateral forelimb (Figure 7, right) in the 6/V/LD group (*p* < 0.01 vs. S/V/V group). Chronic administration of PDM-042 (1 and 3 mg/kg) did not interfere with antiparkinsonian activity of L-DOPA (*p* > 0.05 vs. 6/V/LD group). It is worth noting that few animals could not step during the test because they were dyskinetic when the test was conducted (Figure 7, right). 

#### 2.4.2. PDM-042 Alters Corticostriatal Activity in Dyskinetic Animals

**Figure 8:**
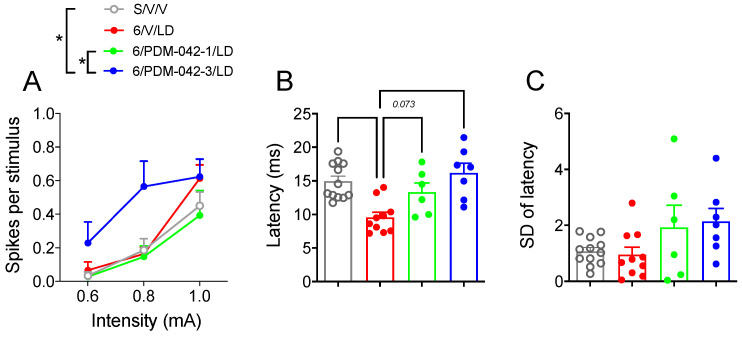
The PDE10A inhibitor PDM-042 alters corticostriatal signaling in LID. (**A**) PDM-042 3 mg/kg but not 1 mg/kg facilitated cortically evoked spike activity in unidentified striatal MSNs recorded in the ipsilateral striatum (* *p* < 0.05 vs. S/V/V and 6/PDM-042-1/LD groups, two-way RM-ANOVA and Holm-Sidak post-hoc test). (**B**) PDM-042 (1 and 3 mg/kg) prevented L-DOPA-induced reduction of spike latency (*p* > 0.05 vs. S/V/V group, one-way RM-ANOVA and Holm-Sidak post-hoc test). (**C**) PDM-042 (1 and 3 mg/kg) had no effect on the standard deviation of spike latency (*p* > 0.05, one-way RM-ANOVA and Holm-Sidak post-hoc test). Data are presented as the mean ± SEM. Results are derived from *n* = 10/12 animals/cells in the S/V/V group, 6/10 animals/cells in the 6/V/LD group, *n* = 4/6 animals/cells in the 6/PDM-042-1/LD group, and *n* = 5/7 animals/cells in the 6/PDM-042-3/LD group.

We attempted to determine the impact of chronic PDM-042 administration on cortically evoked spike activity and spike latency in putative striatal MSNs recorded in the ipsilateral striatum (Figure 8). Two-way RM-ANOVA analysis revealed an effect for both drug treatment (*F*_(3,31)_ = 3.45; *p* < 0.05) and current intensity (*F*_(2,62)_ = 41.97; *p* < 0.0001), but there was no interaction between drug treatment and current intensity (*F*_(6,62)_ = 1.65; *p* > 0.05) on spike probability (Figure 8A). The post-hoc analysis for the main effects of drug treatment revealed that the higher dose of PDM-042 (3 mg/kg) facilitated corticostriatal transmission (*p* < 0.05 vs. S/V/V and 6/PDM-042-1/LD groups; Figure 8A). Because cortical stimulation with 0.6 mA failed to elicit consistent responses in MSNs, we investigated the effects of cortical stimulation on the spike latency at 1 mA current intensity (Figure 8B). One-way ANOVA revealed significant differences in spike latency between treatment groups (*F*_(3,31)_ = 8.81; *p* < 0.001). The post-hoc analysis revealed that dyskinetic animals had a shorter spike latency (*p* < 0.05 vs. S/V/V group). PDM-042 (1 and 3 mg/kg) prevented L-DOPA-induced reduction of spike latency (*p* > 0.05 vs. S/V/V group). There was no difference in the standard deviation of spike latency between groups (*F*_(3,31)_ = 2.48, *p* > 0.05, Figure 8C). 

## 3. Discussion

Although L-DOPA is currently the most widely used pharmacotherapy for treating PD, the development of LID often becomes severe and more harmful than the symptoms of the disease itself. Many studies have tried to elucidate the pathophysiological mechanisms that cause LID to find more effective treatments. This study first investigated behavioral (AIMs and stepping test) and electrophysiological (in vivo single-unit extracellular recordings of striatal MSNs) correlations related to dyskinetic behaviors in parkinsonian rats. Our data demonstrated that MSNs recorded from L-DOPA-treated dyskinetic animals exhibited increased spike probability, decreased spike latency, and a trend toward reduction of the standard deviation of spike latency. Furthermore, there was a moderate to strong correlation between spike probability and both spike latency and standard deviation of spike onset latency. These data suggest that MSNs recorded in the dyskinetic striatum respond to cortical drive with a higher probability. Furthermore, spikes were triggered rapidly (lower onset latency) and more precisely (lower standard deviation) in the dyskinetic striatum. The second cohort of animals was used to investigate the impact of the PDE10A inhibitor PDM-042 on behavioral and electrophysiological correlations of LID in parkinsonian rats. Dyskinesia scoring revealed that the low dose of PDM-042 (1 mg/kg) had an antidyskinetic effect and attenuated peak-dose dyskinesia (60–120 min after L-DOPA administration). The high dose of PDM-042 (3 mg/kg) did not affect peak-dose dyskinesia but prolonged AIMs (120–150 min after L-DOPA administration). PDM-042 (either at low or high doses) did not interfere with the antiparkinsonian efficacy of L-DOPA. Single unit electrophysiological recordings revealed that PDM-042 1 mg/kg did not interfere with cortically evoked responses in striatal MSNs. Interestingly, PDM-042 3 mg/kg facilitated corticostriatal activity and increased cortically evoked responses in striatal MSNs. Both doses of PDM-042 restored MSNs spike onset latency to sham-operated levels. These data suggest that the antidyskinetic effects of PDE10A inhibitors are dose-dependent. Furthermore, the expression of AIMs is likely to correlate with increments in MSN spike activity responsiveness to cortical stimulation. Finally, the facilitation of corticostriatal transmission (spike activity responsiveness to cortical drive) is likely to contribute to the expression of AIMs.

### 3.1. Modulation of AIMs by PDE10A Inhibition

PDE10A inhibitors differ significantly from D2 receptor antagonists in various preclinical behavioral models. They produce low catalepsy suggesting a minimal risk for the induction of extrapyramidal side effects [34,35,36]. The impact of PDE10A inhibitors appears to depend on the activation state of striatonigral and striatopallidal MSNs. For instance, a study demonstrated that inhibition of the PDE10A enzyme could reverse suppressed behavior and even stimulate behavior, depending on the relative activation status of the direct and indirect pathways [34]. Authors demonstrated that when the activity of the direct pathway MSNs was reduced by a D1-like receptor antagonist (inducing catalepsy in the animals), PDE10A inhibitors potentiated catalepsy by enhancing the inhibitory actions of the indirect pathway and acting as a D2-like receptor antagonist. On the other hand, when catalepsy was induced by a D2-type antagonist (haloperidol), PDE10A inhibitors acted as a D1-type agonist, reversing the catalepsy induced in animals [34]. However, the opposite effect was observed with lower doses of haloperidol [22]. 

In our study, the PDE10A inhibitor PDM-042 produced a dose-dependent effect on AIMs and did not interfere with the antiparkinsonian effect of L-DOPA. The low dose of PDM-042 had an antidyskinetic effect and attenuated peak-dose dyskinesia. The high dose of PDM-042 did not affect peak-dose dyskinesia but prolonged the incidence of AIMs. This dose-dependent effect is likely to be related to the fact that PDE10A inhibitors can act concomitantly as D1 dopamine receptor “agonist” and D2 dopamine receptor “antagonists” [34]. In addition, several studies have shown that the inhibition of PDE10A would prefer acting in striatopallidal MSNs, acting mainly as “antagonists” of D2-type receptors [14,19,30,37]. These results suggest that PDE10A inhibitors would increase the activity of the indirect pathway, which would cause less dyskinetic behavior and agrees with the results obtained in the treatment with the low dose of PDM-042. Based on our behavioral data, it is tempting to speculate that treatment with a high dose of PDM-042 may have also increased the activity of the direct pathway, which caused the prolongation of the LID. 

Although PDE10A inhibitors attract considerable interest as a potential target for the treatment of neuropsychiatry [38,39] and movement disorders such as Huntington’s disease (HD) [40] advancing to early clinical safety studies, PDE10A inhibitors from three companies failed to evidence antipsychotic activity in patients with schizophrenia, and a phase-II study conducted by Pfizer showed no efficacy to improve symptoms in patients with HD [41]. In addition, PDEA10 inhibitors unexpectedly revealed a substantial dyskinesia motor side-effect, for which one of the possible explanations include non-optimal dose, titration schedule and, more importantly, the differential non-linear pharmacodynamic interactions with individual co-medications [42]. Nonetheless, this manuscript helps to elucidate the mechanism of these inhibitors and is relevant to improving the translation validity of this class of drugs.

### 3.2. Modulation of Striatal MSN Activity by PDE10A Inhibition

The striatum was the focus of many studies to understand the pathophysiology of LID, but other central nervous system regions are also involved in this dysfunction. A critical area involved in LID is the primary motor cortex (M1) [43]. It has already been shown that abnormal cortical oscillatory activity is related to LID [44,45]. In addition, LID was also related to deficits in M1 neuroplasticity [46]. Through MRI, many studies in patients with PD and LID have demonstrated that cortical activity is hyperactivated or deregulated [9,10,11]. A recent study using a combination of transcranial magnetic stimulation protocols in the primary motor cortex [47] demonstrated that the activity of specific intracortical circuits is upregulated in patients with PD and LID. Unfortunately, the cause and the consequences of the abnormal cortical activity are still poorly understood [43]. Studies in animal models allow us to investigate further the origin of abnormalities in corticostriatal neurotransmission at the molecular and cellular levels. 

A recent publication provided a detailed analysis of chronic PDE10A inhibition in L-DOPA-treated 6-OHDA-lesioned animals [48]. Animals that received chronic administration of the PDE10A inhibitor TP-10 and L-DOPA presented a significant reduction in the expression of PDE10A mRNA in the sensorimotor striatum. Interestingly, this treatment resulted in greater expression of dynorphin and enkephalin in the ipsilateral sensorimotor striatum, indicating that PDE10A regulates gene expression for both markers of striatonigral and striatopallidal (i.e., dynorphin and enkephalin, respectively) [49,50]. Taken together, these results indicate that PDE10A is a crucial modulator of neuroplasticity in response to synaptic input to the striatum [48].

Studies conducted by our group have already demonstrated that inhibition of the PDE10A enzyme with TP-10 could increase the excitability of striatonigral MSNs while having little or no changes in the activity of striatopallidal MSNs [14]. To study the influence of PDE10A inhibition on abnormal corticostriatal activity during dyskinetic behaviors, we performed in vivo single-unit extracellular recordings to address the impact of cortical stimulation on the activity of striatal MSNs. We found that the PDE10A inhibitor PDM-042 attenuated LID, did not interfere with cortically evoked spike probability, and restored spike latency to control levels when administered at 1 mg/kg to dyskinetic rats. On the other hand, PDM-042 prolonged the incidence of LID, facilitated cortically evoked spike probability, and restored spike latency to control levels when administered at the dose of 3 mg/kg. Although this study did not dissect the effects of PDM-042 on the direct and indirect pathway, the results indicate that PDE10A activity is involved with temporal MSNs coding of glutamatergic information from the motor cortex. Based on the data presented here, it is likely that the antidyskinetic effects of PDE10A inhibitors depend on their modulation of MSNs activity to cortical drive. Inhibition of the PDE10A enzyme at levels that produces antidyskinetic activity is expected to facilitate cyclic nucleotide signaling and restore the balance between DA and glutamate systems. Doses of PDE10A inhibitors that facilitate corticostriatal transmission are likely to prolong L-DOPA effects, leading to increased duration of LID. Thus, the normalization of glutamatergic transmission using PDE10A inhibitors may be related to the impact of PDM-042 in LID. 

## 4. Materials and Methods

### 4.1. Animals

Adult male Sprague-Dawley rats (weighing 200–250 g at the beginning of the experiment) were grouped in boxes with 3 or 4 animals, with access to food and water ad libitum and kept under controlled conditions of temperature (22 ± 2 °C), air exchange (15–20 exchanges/h) and light–dark cycles (12 h light/12 h dark). 

### 4.2. Drugs

L-DOPA (Cayman Chemical Company, Ann Arbor, MI, USA) and benserazide (Sigma-Aldrich, Saint Louis, MO, USA) were dissolved in sterile 0.9% saline solution and administered subcutaneously (s.c., 2 mL/kg). The PDE10A inhibitor PDM-042 (synthesized at Mochida Pharmaceutical Co., Tokyo, Japan) was dissolved in 0.9% saline containing 0.5% methylcellulose and administered by gavage (5 mL/kg). All other reagents were of the highest grade commercially available. The pharmacological characterization of PDM-042 ((*E*)-4-(2-(2-(5,8-dimethyl-[1,2,4]triazolo [1,5-*a*]pyrazin-2-yl)vinyl)-6-(pyrrolidin-1-yl)pyrimidin-4-yl)morpholine) was described before [20]. PDM-042 demonstrated high brain penetration (striatum/plasma ratio = 6.3). The occupancy rate of PDE10A for PDM-042 at its ED50 (0.44 mg/kg) was 39%. The occupancy rates of PDE10A at the doses used of 1 and 3 mg/kg were 66% and 86.6%. Furthermore, PDM-042 showed good oral bioavailability (33%) [20]. Thus, PDM-042 is a highly powerful, selective, orally active, and brain penetrant PDE10A inhibitor.

### 4.3. 6-OHDA Lesion

For the induction of the experimental parkinsonism, stereotactic surgery was performed for microinjection of the neurotoxin 6-OHDA (Sigma-Aldrich, Saint Louis, MO, USA) into the right MFB [32]. For efficacy and selectivity of the lesion, the animals were treated with imipramine (Alpha Aesar, Tewksbury, MA, USA; 20 mg/kg; intraperitoneal), a noradrenaline and serotonin reuptake inhibitor, 30 min before 6-OHDA infusion, to prevent 6-OHDA from being taken up by noradrenergic terminals. Subsequently, the animals were anesthetized with ketamine (Dopalen, Sespo Indústria e Comércio Ltda, Paulínia, SP, Brazil; 70 mg/kg; intraperitoneal) and xylazine (Anasedan, Sespo Indústria e Comércio Ltda, Paulínia, SP, Brazil; 10 mg/kg; intraperitoneal) and fixed in a stereotaxic apparatus (Insight Equipamentos^®^, Ribeirão Preto, SP, Brazil). After exposing the animal’s skull, the coordinates of the right MFB were taken for unilateral 6-OHDA microinjection (AP: −4.3 mm, ML: +1.6 mm, and DV: −8.3 mm, from bregma [31]. Subsequently, 4 µL (at a rate of 0.5 µL/min) of 6-OHDA were microinjected into 0.9% saline solution containing 0.1% ascorbic acid or vehicle only (sham-operated control animals). Finally, the incision was sutured, and the animal was placed in a heated box under a thermal blanket for recovery from anesthesia.

### 4.4. Treatment Groups and Drug Administration

Drug treatments started 4 weeks after the surgery. All putative 6-OHDA-lesioned animals (i.e., animals with a 6-OHDA lesion that met the inclusion criterion of five or fewer forelimb adjusting steps) were included. The first cohort of animals was used to investigate how MSNs respond to motor cortex stimulation during LID. A group of 6-OHDA-lesioned animals was treated with L-DOPA (5 mg/kg combined with benserazide 12.5 mg/kg, s.c.) once a day, from Monday to Friday [51] for three weeks (6/LD, *n* = 15). A group of 6-OHDA-lesioned (6/V, *n* = 17) and a group of sham-operated animals (S/V, *n* = 13) were treated with vehicle. The second cohort of animals was used to investigate the effects of the PDE10A inhibitor PDM-042 on LID. Only animals that developed AIMs (*n* = 21) at the end of the first week of L-DOPA treatment were included in the subsequent treatments. During the second and third week of treatment, dyskinetic animals received vehicle (0.9% saline solution containing 0.5% methylcellulose) (6/V/LD, *n* = 9) or the PDE10A inhibitor PDM-042 (gavage) at the doses of 1 mg/kg (6/PDM-042-1/LD, *n* = 7) and 3 mg/kg (6/PDM-042-3/LD, *n* = 5) 1 h before the administration of L-DOPA. A group of sham-operated animals (S/V/V, *n* = 10) was treated with vehicle.

In all experiments, forelimb akinesia was assessed once a week (Tuesday) before vehicle/drug administration and at 60 min post-L-DOPA injection to detect possible interference of tested drugs with the antiparkinsonian efficacy of L-DOPA. All rats were evaluated three times per week for the presence of LID (Wednesday–Friday).

### 4.5. Stepping Test and Lesion Assessment

Before the beginning of the stepping test, the animals were trained for 3 days to become familiarized with the researcher and the test. The experimenter held the rat at a 45-degree angle during the test, immobilizing its hind limbs and allowing only one of the front limbs to rest on the surface. With the right or the left paw resting on the surface, the rat is dragged (90 cm in 4 s) in the forehand direction. The number of steps taken with each paw in each direction was quantified [17,18]. Animals that presented 5 steps or less with the paw contralateral to the lesion were treated as putative 6-OHDA-lesioned animals [52]. The stepping test was performed before, 2, and 4 weeks after stereotactic surgery to assess the contralateral forelimb akinesia. The test was also performed on the third week (Tuesday) of chronic treatment 60 min after the administration of L-DOPA to evaluate if PDM-042 interferes with L-DOPA’s antiparkinsonian efficacy. The lesion was confirmed histologically at the end of the behavioral tests by tyrosine hydroxylase (TH) immunohistochemistry, as previously described by our group [32]. It is important to note that when the dopaminergic lesion is not complete, the results of the stepping test will not reach the minimum number of steps adopted in this study [32,33].

### 4.6. Abnormal Involuntary Movements (AIMs)

Abnormal involuntary movements (AIMs) were videotaped (2 min) at 30 min intervals (30–180 min) post-vehicle or L-DOPA injection. Scores were given over 1 min epochs and classified as axial, limb, and orolingual. The severity of AIM subtypes was scored using a standard scale (0 = absent; 1 = occasional; 2 = frequent with many interruptions; 3 = frequent but interrupted by an external stimulus; and 4 = continuous) [51,53,54]. Additionally, scores for the amplitude of axial (0 = absent; 1 = consistent lateral deviation of head and neck at approximately 30° angle; 2 = deviation of head and neck, 30° < angle ≤ 60°; 3 = lateral deviation of head, neck, and upper trunk, 60° < angle ≤ 90°; 4 = torsion of the head, neck, and trunk at >90° angle making rats lose balance), limb (0 = absent; 1 = little involuntary movements of the distal forelimb; 2 = movements of low amplitude causing translocation of both distal and proximal forelimb; 3 = involuntary movements of the whole limb including shoulder muscles; 4 = strong limb and shoulder movements with similarity to ballism) and orolingual (0 = absent; 1 = little involuntary movements of the orolingual muscles; 2 = movements of high amplitude causing tongue protrusion) were assigned as previously described [55]. Partial scores such as 0.5, 1.5, 2.5, and 3.5 were assigned to increase the sensitivity of the rating. Severity and amplitude AIMs scores were multiplied on each monitoring period (i.e., 30, 60, 90, 120, and 180 min) and then summed, giving a total AIM score subtype for each testing day. Temporal scores for each AIM subtype are presented as the sum of scores obtained on Wed, Thu, and Fri.

### 4.7. Electrophysiology

To maximally preserve the circuitry involved in mediating corticostriatal transmission and assess potential correlations between behavioral and electrophysiological outcomes, in vivo electrophysiological recordings of cortically evoked activity were performed in the same cohort of animals/treatment groups from behavioral studies. Drug treatment groups were compared to their respective control groups. On the recording day, rats were anesthetized with urethane, and a bipolar stimulating electrode was implanted in the primary motor cortex (AP: +3.0 mm, ML: 2.5 mm, and DV: 2.0 mm) ipsilateral to the parkinsonian striatum. A microelectrode manufactured from glass capillary (2.0 mm diameter, filled with NaCl 2M solution, WPI) was slowly introduced into the striatum (AP: +0.75 mm, ML: 3.5 mm, and DV: 2.5 to 7.0 mm) with a micromanipulator (MO-8, Narashige) while low-frequency electrical stimuli (0.5 Hz, 0.5 ms) were applied to the ipsilateral motor cortex. Low-frequency stimuli (0.5 Hz, 0.5 ms) were applied to the motor cortex to mimic the natural occurrence of glutamatergic activity in anesthetized animals [14,56,57]. Since the low-frequency pulses used in this study stimulate glutamatergic activity that converges to the striatal MSNs, this cortical stimulation protocol simulates the glutamatergic activity that occurs naturally in the basal ganglia responsible for generating action potentials in the MSNs [58]. When a neuron responded to cortical stimuli with a short latency (monosynaptic, 5–15 ms), the probability of firing evoked by stimulation of the motor cortex was assessed for 50 cortical stimuli, in 3 different intensities (600, 800, and 1000 µA). The activity of each isolated neuron was amplified (IR-183, Cygnus Technology Inc., Southport, NC, USA) filtered (8 kHz for low-pass and 400 Hz high-pass), digitized (20 kHz), and recorded on a computer integrated with a data acquisition system (Digidata 1550B4, Molecular Devices). Striatal neurons exhibiting action potentials characteristic of cholinergic or nitrergic neurons (firing rates of 1 to 4 Hz) or parvalbuminergic (fast-spiking interneurons, FSIs; which respond to low-intensity cortical stimulation with short action potentials) were not grouped with the MSNs. The responsiveness to cortical stimulation was assessed in multiple striatal neurons 30 to 180 min after the last injection of vehicle/drug.

Cortically responsive putative MSNs exhibited an action potential duration of >0.95 ms. We attempted to classify cortically responsive putative MSNs neurons as striatonigral or striatopallidal MSNs using antidromic stimulation of the SNr. Using the collision test, approximately 5% of putative MSNs were identified as SNr+. We often found striatal neurons that were unresponsive to cortical stimulation, but they did respond to antidromic stimulation of SNr. Because it was impossible to execute the collision test, these cells were excluded from the study. Because the numbers of identified striatonigral MSNs were not sufficient for statistical comparisons, data from striatonigral (identified via antidromic stimulation of the SNr) and unidentified MSNs were pooled for this analysis.

### 4.8. Tissue Processing

Rats were deeply anesthetized with urethane and rapidly perfused (10 mL/min) transcardially with 150 mL of artificial cerebrospinal fluid (4 °C, pH 7.4). Brains were immediately removed and postfixed for 90 min in freshly prepared fixative (4% paraformaldehyde dissolved in 0.15 M sodium phosphate buffer, pH 7.4) as previously described [59]. Brains were cryoprotected in 30% sucrose in phosphate-buffered saline (4 °C), frozen with dry ice, and stored at −80 °C. Coronal sections (50 µm) were processed on a freezing microtome throughout the rostrocaudal extent of the motor cortex (+3.72 mm to +2.52 mm), striatum (+2.16 mm to −0.84 mm), and substantia nigra (−4.44 mm to −6.72 mm) [31].

### 4.9. Tyrosine Hydroxylase Immunohistochemistry and Lesion Assessment

Immunostaining was performed on free-floating sections of the substantia nigra compacta with standard avidin–biotin protocols [50]. Briefly, sections were incubated overnight at room temperature with rabbit anti-TH primary antibody (1:4000, Pel-Freez LLC, Rogers, AR, USA), followed by 90 min of incubation with anti-rabbit biotinylated secondary antibody (1:500, Vectastain, Vector Laboratories, Newark, CA, USA). Sections were developed using diaminobenzidine as the chromogen. The slices were mounted on slides and coverslipped for microscopic observations. The number of TH positive cells was counted in four sections per animal between bregma −5.28 and −6.00 mm using the ImageJ software (National Institute of Health, USA). Only cells containing visible nuclei and at least one emerging dendritic process were considered positive for TH [60,61].

### 4.10. Data Analysis

The stepping test, the scores applied for the AIMs, and the electrophysiological data were evaluated according to previously established criteria [24]. The statistical significance of drug-induced effects on the stepping test, AIMs, and neuronal activity were determined using *t*-tests and one-way and two-way ANOVAs in agreement with previous publications [53,62,63,64]. Holm-Sidak’s post-hoc test determined which group(s) contributed to ANOVA’s overall effects or interactions. Pearson’s correlation analysis was conducted to investigate the relation between spike probability and spike latency. A probability of *p* < 0.05 was considered to determine significant differences. All statistical data were analyzed using GraphPad Prism software (Version 9.0—GraphPad Software, San Diego, CA, USA).

## 5. Conclusions

In conclusion, our results demonstrated that the effects of PDM-042 in LID and corticostriatal transmission are dose-dependent. An antidyskinetic dose of PDM-042 had no effect on corticostriatal activity, whereas a dose of PDM-042, which prolonged the duration of L-DOPA effects and LID, was associated with a facilitation of corticostriatal activity. We believe the data presented here will help elucidate the neurophysiological mechanisms related to abnormal corticostriatal transmission in LID.

## Data Availability

Data is contained within the article.
